# Low Expression of Phosphodiesterase 2 (PDE2A) Promotes the Progression by Regulating Mitochondrial Morphology and ATP Content and Predicts Poor Prognosis in Hepatocellular Carcinoma

**DOI:** 10.3390/cells12010068

**Published:** 2022-12-23

**Authors:** Lin Chen, Jinchi Zhou, Zifeng Zhao, Yuhan Zhu, Jinliang Xing, Jiaze An, Xu Guo

**Affiliations:** 1 State Key Laboratory of Cancer Biology and Department of Physiology and Pathophysiology, Fourth Military Medical University, Xi’an 710032, China; 2 Department of Plastic Surgery, Xijing Hospital, Fourth Military Medical University, Xi’an 710032, China; 3 Department of Gastroenterology, The Second Affiliated Hospital of Fourth Military Medical University, Xi’an 710032, China; 4 Department of Hepatobiliary Surgery, Xijing Hospital, Fourth Military Medical University, Xi’an 710032, China

**Keywords:** phosphodiesterase 2 (PDE2A), hepatocellular carcinoma (HCC), mitochondrial morphology, ATP content, prognosis

## Abstract

Phosphodiesterase 2 (PDE2A) modulates the levels of cAMP/cGMP and was recently found to be involved in mitochondria function regulation, closely related to multiple types of tumor progression. This study aimed to estimate the prognostic significance and biological effects of PDE2A on hepatocellular carcinoma (HCC). We comprehensively analyzed the PDE2A mRNA expression in HCC based on The Cancer Genome Atlas (TCGA) database and investigated the effects of PDE2A on the proliferation and metastatic capacity of HCC cells. PDE2A was downregulated in 25 cancer types, including HCC. Lower PDE2A expression was a protective factor in HCC and was negatively associated with serum AFP levels, tumor status, vascular invasion, histologic grade, and pathologic stage of HCC. Moreover, tumors with low PDE2A expression displayed a decreased immune function. Then, the ROC curve was used to assess the diagnostic ability of PDE2A in HCC (AUC = 0.823 in TCGA and AUC = 0.901 in GSE76427). Patients with low PDE2A expression exhibited worse outcomes compared with those with high PDE2A expression. Additionally, GO functional annotations demonstrated the involvement of PDE2A in the ECM organization, systems development, and ERK-related pathways, indicating that PDE2A might regulate HCC growth and metastasis. The in vitro experiments confirmed that overexpression of PDE2A inhibited proliferation, colony formation, migration, and invasion in two HCC cell lines (HLF and SNU-368), while inhibition of PDE2A has the opposite results. The mechanism of PDE2A’s effect on HCC cells is attributed to the change of mitochondrial morphology and ATP content. These data demonstrated that PDE2A closely participated in the regulation of HCC proliferation and metastasis and can be used as a predictive marker candidate and a potential therapeutic target for HCC.

## 1. Introduction

Hepatocellular Carcinoma (HCC) is the most common type of primary liver cancer, accounting for 80% of liver cancers [1]. According to the latest statistics, approximately 906,000 new cases and 830,000 deaths were reported worldwide in 2020 [2]. Unfortunately, most HCC patients are diagnosed at late stages, and their prognosis remains unfavorable. The mortality to incidence was up to 0.95 as previous reported [3], showing a very poor prognosis. At present, through ultrasound, serum alpha-fetoprotein (AFP) detection, and CT scan, HCC could be diagnosed, but it is often misdiagnosed [4]. Although various biomarkers have been applied to HCC, such as AFP and Glypican-3, their reliability and sensitivity remains controversial. Other new tumor biomarkers have been proposed, such as osteopontin (OPN), vascular endothelial growth factor (VEGF), angiopoietin 2 (ANG-2), Golgi protein 73 (Gp-73), insulin growth factor-1 (IGF-1), hepatic growth factor (HGF), and c-MET among others [5], but they are still a long way from clinical use. Thus, it is urgent to comprehensively depict the signatures of HCC, identify novel molecular biomarkers, and search for effective therapeutic targets.

Phosphodiesterases (PDEs), through degradation of guanosine 3′, 5′-cyclic monophosphate (cGMP) and adenosine 3′, 5′-cyclic-monophosphate (cAMP), two important second messengers [6], significantly participate in the regulation of many cell signalings [7]. Many pathologies, such as oxidative stress, neurodegeneration, inflammation, and cancer, were dramatically affected by PDEs, by controlling those intracellular signaling pathways [7]. PDE2A, also called cGMP-stimulated PDE (cGS-PDE), was first identified in 1971 [8]. PDE2A functions as a regulator for cAMP/cGMP levels. Recently, several studies found that PDE2A also regulates mitochondrial morphology [9], mitochondrial clearance [10], and protein phosphorylation [11]. Moreover, PDE2A plays a key role in cancer progression, including colorectal cancer [11], melanoma [12], and so on. Li SZ et al. found that PDE2A is significantly downregulated in glioma and overexpression it decelerated glioma progression [13]. Frolov A et al. found that PDE2A is significantly downregulated in gastrointestinal stromal tumors [14]. In HCC, Guihua Rao et al. found PDE2A is one of the stem cell index-related gene sets, and combined with other six genes are a good prognostic model. Zhang J et al. constructed a predictive model including PDE2A and the other six genes, successfully predicting the survival rate of cholangiocarcinoma, HCC, and pancreatic adenocarcinoma patients [15]. Lajos Pusztai et al. found that PDE2A is one of the targets of exisulind, a selective apoptotic antineoplastic drug, in metastatic breast cancer [16]. Additionally, it has been reported that a specific radioligand for the imaging of PDE2A via positron emission tomography (PET) could be used as an effective way to detect the expression change in the brain [17]. Moreover, PDE2A is indispensable for mouse liver development and hematopoiesis. The PDE2A knockout mouse has impaired liver and hematopoietic development, and the size and quality of the liver is much smaller and lower than in normal embryonic mice [18]. However, the biological effects and the prognostic values of PDEA2 for HCC are not completely clarified yet. 

Therefore, in the present study, we comprehensively analyzed the correlation between PDE2A and HCC using RNA sequencing data from The Cancer Genome Atlas (TCGA). We also found that PDE2A closely participated in the proliferative and metastatic processes of HCC cells in vitro and can be used as a predictive factor candidate and a potential therapeutic target for HCC.

## 2. Materials and Methods

### 2.1. Processing of RNA Sequencing (RNA-Seq) Data 

The RNA-seq profiles of pan-cancer data from TCGA that included 371 HCC cases (TCGA-LIHC) were retrieved from the GDC Data Portal accessed on 27 March 2022. (https://portal.gdc.cancer.gov). Raw files were first processed using the “Limma” R package and genes were annotated by the Ensembl human genome browser (http://asia.ensembl.org/index.html (accessed on 19 December 2022)). Meanwhile, the clinical data in TCGA were extracted from the cBioportal at 7 April 2022 (http://www.cbioportal.org/) website, including molecular status, survival data (e.g., status and overall survival time), and other basic information (e.g., age, gender). The expression of PDE2A was normalized by log2 [transcripts per kilobase million (TPM) + 1] transformation.

### 2.2. Detection of Differentially Expressed Genes (DEGs) and Gene Set Enrichment Analysis (GSEA)

Based on the expression levels of PDE2A, the patients of TCGA were classified into two groups: PDE2A high and low expression groups. Then, the mean normalized readcounts were used to identify the DEGs between these groups by the “DESeq2” package. Hochberg and Benjamini’s approach were used to control the false discovery rate (FDR) of *p*-values. A log fold change (logFC) > 1.5 and FDR < 0.05 were set as the screening criteria. To analyze the enrichment and functional annotation, we uploaded all DEGs to the Metascape database (http://metascape.org (accessed on 19 December 2022)) [19]. Further, the GSEA of these DEGs was conducted using the “clusterProfiler” R package. Significant differences in the clusters were set as FDR < 0.25 and *p* < 0.05, respectively.

### 2.3. Protein–Protein Interaction (PPI) Analysis

We used the STRING protein interaction database to analyze the DEGs PPI network with a confidence score > 0.7 (https://string-db.org/ (accessed on 19 December 2022), Organism: Human). Cytoscape was applied for network visualization. By counting the interactions between each network node with others, hub proteins were identified according to the scale-free property of interaction networks. Cytoscape was also applied for network module analysis by using MCODE with default parameters (Degree Cutoff: 2, NodeScore Cutoff: 0.2, K-Core: 2, and Max. Depth: 100).

### 2.4. Survival Analysis

To examine the prognostic value of PDE2A in HCC, we performed survival analysis of overall survival (OS), disease-free survival (DFS), and progression-free interval (PFI). The HCC cases were divided into two groups based on the median of PDE2A mRNA expression (Median of log2 [TPM + 1] = 0.915): PDE2Ahigh and PDE2Alow groups. Multivariate and univariate Cox regressions and Kaplan–Meier curve were used for the survival analysis.

### 2.5. Associations of PDE2A with Immune Cell Infiltration in HCC

The enrichment of the infiltrated immune cells, such as NK 56+ cells, NK 56-cells, cytotoxic cells, macrophages, B cells, CD8+ T cells, T follicular helper (Tfh) cells, T central memory (Tcm) cells, Tgamma delta (Tgd) cells, regulatory T cells (Treg) cells, type 17 Th (Th17) cells, type 2 Th (Th2) cells, type 1 Th (Th1) cells, T helper (Th) cells, T cells, dendritic cells (DCs), plasmacytoid DCs (pDCs), activated DCs (aDCs), immature DCs (iDCs), T effector memory (Tem) cells, eosinophils, neutrophils, mast cells, and natural killer (NK) cells, were quantified based on the corresponding gene expression profile to in the previous literature [20], and were then evaluated by the single sample GSEA method using the “GSVA” package. Then, Spearman rank test was carried out to evaluate the association of PDE2A expression with the infiltration of immune cells. The Wilcoxon rank sum test was conducted to compare the immune cell infiltration between PDE2A^high^ and PDE2A^low^ groups.

### 2.6. Immunohistochemistry (IHC) Staining

The study was approved by the Ethics Committee of Xijing Hospital, the Fourth Military Medical University, China. Ten cases of para-tumor, T1, T2, and T3 stage HCC clinical samples were collected from Xijing hospital, the Fourth Military Medical University, China. All patients agreed to participate in this study, and written informed consent was obtained from all subjects. First, 4 μm thick sections were cut from paraffin-embedded tissue fixed by 4% paraformaldehyde. After being deparaffinized with xylene and rehydrated with ethanol, the sections were then heated in a water bath for 30 min with a pH 9.0 ethylenediaminetetraacetic acid buffer for antigen retrieval, then the endogenous peroxidases were inactivated by 3% H_2_O_2_ for 15 min. Then, the sections were blocked by 5% bovine serum albumin for 30 min at 28 °C and the primary antibody of PDE2A (55306-1-AP, Proteintech Group, Wuhan, China) was incubated at 4 °C overnight at a dilution of 1:200. Subsequently, the sections were incubated with a second antibody (Maixin biotechologles, Fuzhou, Fujian, China), followed by 3,3-diaminobenzidine (DAB) staining. The results were viewed using a light Olympus microscope. Staining intensity was quantified independently by two pathologists in a double-blinded fashion.

### 2.7. Overexpression and Knockdown of PDE2A in HCC Cell Lines

Total RNA was extracted from human 293T cells, then reverse transcribed to cDNA. The PDE2A gene was amplified using the above-synthesized cDNA. The PCR-amplified PDE2A gene was cloned into the pLV3-CMV vector. After verification by sequencing, PDE2A plasmid and empty vector (EV) plasmid with lentivirus helper plasmid pLP1, pLP2, and pVSVG were transfected into 293T cells to produce lentivirus. The HLF and SNU-368 HCC cell lines were infected with the PDE2A overexpression lentivirus with an empty vector (EV) as control. Puromycin was used to screen positive cells. The screened PDE2A-overexpressed cells were amplified to verify the PDE2A expression by RT-qPCR, then used in the downstream experiments. For knockdown of PDE2A, the siRNA was transfected into SNU-368 and HLF cells by Lipofectamine™ 2000 (Invitrogen, Waltham, MA, USA). The sequences of PDE2A siRNA are: Sense: 5′-3′: GGAGCUGAUCUACAAAGAATT; Anti-sense: 5′-3′: UUCUUUGUAGAUCAGCUCCTT; and the sequences of the negative control are: Sense: 5′-3′: UUCUCCGAACGUGUCACGUTT; Anti-sense: 5′-3′: ACGUGACACGUUCGGAGAATT. Cells were cultured in Dulbecco’s modified Eagle’s medium (DMEM, Gibco, Waltham, MA, USA) under 5% CO_2_ at 37 °C. The primers used are as follows: PDE2A: Forward: 5′-3′ CGCCCATTCTCTCCTATACAAA; Reverse: 5′-3′GAGACCTTCATGTGGTACATCA. GAPDH: Forward: 5′-3′: GCACCGTCAAGGCTGAGAAC; Reverse: 5′-3′: TGGTGAAGACGCCAGTGGA. The antibodies used were anti-PDE2A (55306-1-AP, Proteintech Group, Wuhan, China) and anti-β-Actin (AC026, ABclonal, Wuhan, China).

### 2.8. Cell Proliferation Assays

We employed the MTS ((3-(4,5-dimethylthiazol-2-yl)-5-(3-carboxymethoxyphenyl)-2-(4-sulfophenyl)-2H-tetrazolium)) assay (Promega Corporation, Madison, WI, USA) to evaluate the proliferative capacity of the indicated cells. The amount of formazan was measured by the spectrophotometer at 490 nm. Briefly, after adding 10 μL MTS reagent into cells cultured in a 96-well plate and 3 h of incubation at 37 °C, we measured the absorbance at 490 nm using the TECAN GENIOS microplate reader (Tecan Group Ltd., Männedorf, Switzerland).

### 2.9. Colony Formation Assay

We performed a colony formation assay to assess the single cell proliferation viability under PDE2A overexpression or knockdown [21]. After placing samples in a 6-well plate with a density of 0.5 × 10^3^ cells/well and 3 weeks of incubation at 37 °C, HLF and SNU-368 cells with indicated treatments were washed using PBS, then fixed using pre-cooled (1:1) methanol/acetone for 15 min at −20 °C. Next, after 5-min staining using crystal violet (0.1%), the formed colonies were observed under a light microscope.

### 2.10. Measurement of Cell Migration and Invasion

The cell migration and invasion assays were performed as previously described [22] for the determination of the migrative and invasive abilities of PDE2A overexpressed or down-expressed cells. The wound-healing assay was performed to evaluate the migration of HCC cells. First, after seeding of PDE2A-overexpressed and control HLF and SNU-368 cells (2 × 10^5^ per well) into a 6-well plate and 24-h incubation, a 1 mL-pipette tip was used to generate the wounds at the bottom of the wells. After PBS wash, photographs of the scratched wounds in each group were taken using a light Olympus microscope at 0 and 48 h after wounding. Image J was used to calculate migration rates.

To investigate the effects of PDE2A on cell invasion, we used 24-well Corning BioCoat Matrigel Invasion Chambers (Corning Inc., New York, NY, USA). PDE2A-overexpressed and control HLF and SNU-368 cells were resuspended in serum-free media and added to the upper chambers. The FBS (10%)-contained medium was added in the bottom chambers. After 24h of culture, 15 min of fixation using ice-cold ethanol at −20 °C, and 5 min of staining using 0.1% crystal violet at room temperature, the invaded cells were observed and counted using a light microscope and ImageJ, respectively.

### 2.11. Mitochondrial Imaging and ATP Detection

MitoTracker Red FM (Invitrogen, Waltham, MA, USA) were used to monitor mitochondrial morphology in living cells according to the manufacturer’s instructions. ATP were detected by ATP-Red1(yk-HY-U00451, MedChemExpress, Shanghai, China). Then, cells were viewed with an Olympus FV 1000 laser-scanning confocal microscope (Olympus Corporation, Tokyo, Japan). For morphometric analysis, the length of mitochondria was measured using the ImageJ software (NIH, Bethesda, MD, USA). The ATP were quantified by the average fluorescence intensity analyzed by ImageJ software.

### 2.12. Statistical Analysis

For the evaluation of the association of PDE2A expression with HCC clinicopathological characteristics, such as the alpha-fetoprotein (AFP) level, tumor status, vascular invasion, histologic grade, pathologic stage, and T stage, we carried out the logistic regression analysis. To evaluate the value of PDEA2 for the prediction of HCC diagnosis, we conducted receiver operating characteristic (ROC) analyses. GSE76427 database was downloaded from https://www.ncbi.nlm.nih.gov/geo/query/acc.cgi?acc=GSE76427 accessed on 19 September 2022. A nomogram was constructed by using the R package “rms”. To assess the nomogram’s prognostic accuracy, calibration and ROC curves were performed. The clinical utility of PDE2A was assessed by using decision curve analysis. For the decision curve, the horizontal axis indicates the threshold probability with a range of 0.0 to 1.0. The vertical axis indicates the clinical net benefit values. A larger area under the decision curve suggests a better clinical utility. Data were analyzed using GraphPad Prism version 9 (GraphPad Software, Inc., San Diego, CA, USA). The student’s *t*-test was used to compare differences between the PDE2A-overexpressed and EV groups. A *p* < 0.05 was considered statistically significant. Values are expressed as means ± SEM.

## 3. Results

### 3.1. Decreased PDE2A Expression in HCC

**Figure 1:**
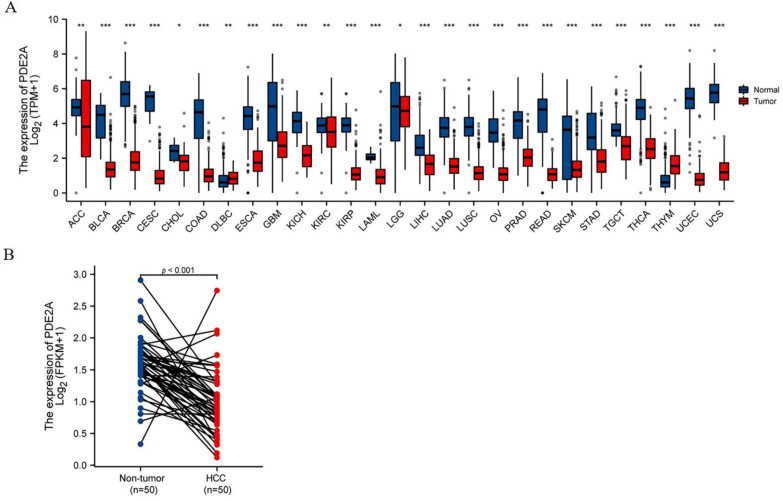
PDE2A mRNA expression profiles. (**A**) Pan-cancer analysis of PDE2A mRNA expression based on TCGA database. ns, *p* ≥ 0.05; * *p* < 0.05; ** *p*< 0.01; *** *p* < 0.001. (**B**) The comparison of PDE2A expression between HCC and para cancer non-tumor tissue in paired HCC samples. ACC: Adrenocortical Cancer; BLCA: Bladder Cancer; BRCA: Breast Cancer; CESC: Cervical Cancer; CHOL: Bile Duct Cancer; COAD: Colon Cancer; DLBC: Large B-cell Lymphoma; ESCA: Esophageal Cancer; GBM: Glioblastoma; KICH: Kidney Chromophobe; KIRC: Kidney Clear Cell Carcinoma; KIRP: Kidney Papillary Cell Carcinoma; LAML: Acute Myeloid Leukemia; LGG: Lower Grade Glioma; LIHC: Liver Cancer; LUAD: Lung Adenocarcinoma; LUSC: Lung Squamous Cell Carcinoma; OV: Ovarian Cancer; PRAD: Prostate Cancer; READ: Rectal Cancer; SKCM: Melanoma; STAD: Stomach Cancer; TGCT: Testicular Cancer; THCA: Thyroid Cancer; THYM: Thymoma; UCEC: Endometrioid Cancer; UCS: Uterine Carcinosarcoma.

Since PDE2A plays an important role in regulating cAMP/cGMP levels and mitochondrial functions, which are closely related to tumor progression, we analyzed the mRNA expression of PDE2A in TCGA pan-cancer datasets. PDE2A expression levels of tumor tissues were significantly lower than the corresponding normal tissues in 25 of 27 cancer types (Figure 1A). Especially in HCC, PDE2A was also substantially decreased in tumor tissues than in non-tumor tissues, either in non-paired samples or paired tissues (*p* < 0.001, Figure 1A LIHC and Figure 1B). Interestingly, the only two types of cancers with high PDE2A expression were Lymphoid Neoplasm Diffuse Large B-cell Lymphoma (DLBC) and Thymoma (THYM), both immune system cancers.

### 3.2. PDE2A-Associated DEGs and Functional Annotation in HCC

**Figure 2:**
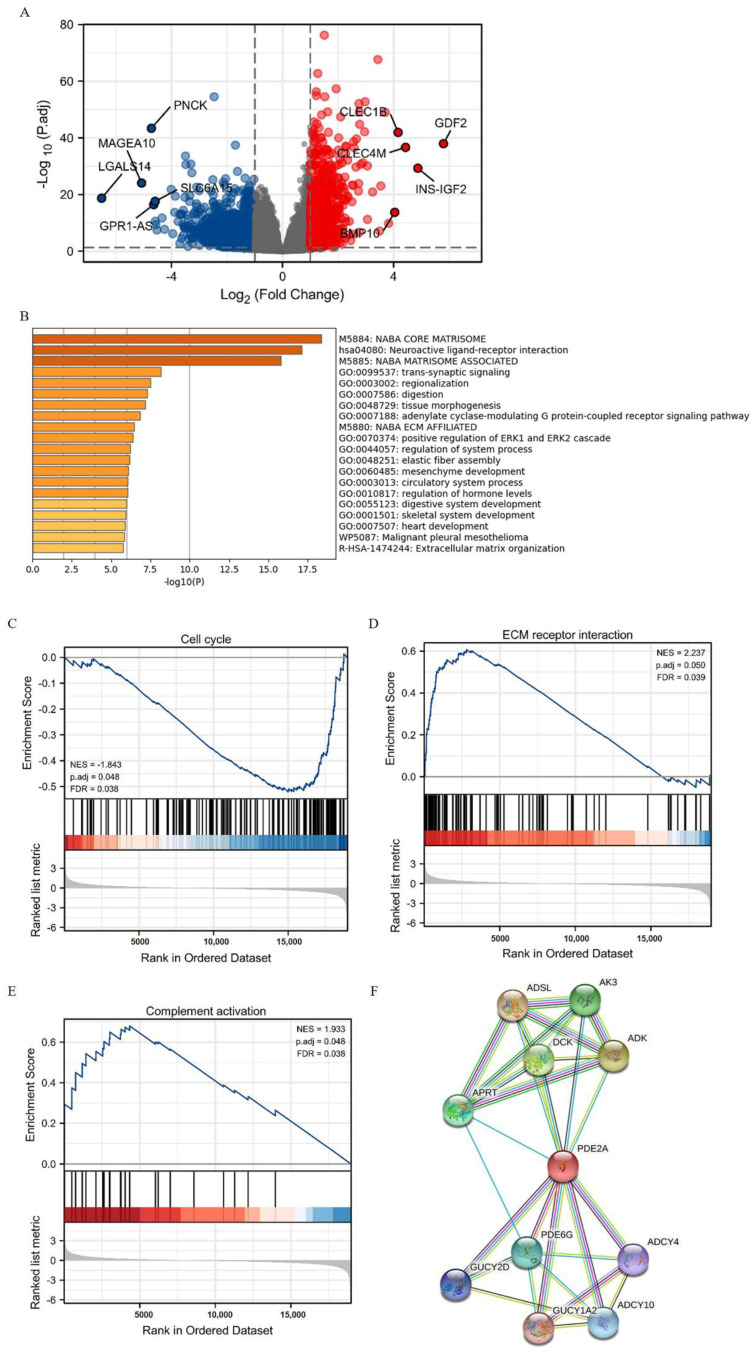
The identification and functional annotation of PDE2A-associated differentially expressed genes (DEGs) in HCC based on the median level of PDE2A. (**A**) Volcano Plot of PDE2A-associated DEGs between high- and low-PDE2A expression groups based on the median PDE2A level. (**B**) Top 20 pathways of functional annotation of DEGs between high- and low-PDE2A expression groups according to the Metascape database. (**C**–**E**) Gene Set Enrichment Analysis (GSEA) of differentially expressed mRNAs between high- and low-PDE2A expression groups. (**F**) PPI (protein-protein interaction) analysis of PDE2A-associated DEGs between high- and low-PDE2A expression groups.

Based on the PDE2A expression levels, the HCC patients of TCGA were classified into two groups: PDE2A high and low expression groups. Next, we compared the expression of the lncRNAs, miRNAs, and mRNAs between these two groups. We totally identified 876 DEGs (fold change ≥ 1.5, adjusted *p* < 0.05) in the high PDE2A group when compared with the low PDE2A group, including 343 lncRNAs, 290 downregulated and 43 upregulated; 1 downregulated miRNA; and 532 mRNAs, 362 downregulated and 170 upregulated (Figure 2A).

Further, we used the functional annotation using the Metascape database to evaluate the function of PDE2A-associated DEGs in HCC. We found that the GO terms associated with ECM (extracellular matrix) organization and tumor metastasis were enriched (M5884: NABA CORE MATRISOME, M5880: NABA_ECM_AFFILIATED, and R-HAS-1474244: Extracellular matrix organization) (Figure 2B). In addition, PDE2A was closely related to the ERK pathway (GO: 0070374), contributing to the control of many cellular processes, including cell proliferation, angiogenesis, development, differentiation, oncogenesis, and cell cycle. Moreover, the GSEA results showed that the PDE2A-related DEGs in clusters were significantly associated with cell proliferation, mitosis and cell cycle (Figure 2C and Supplementary Figure S1(A1–A9)), tumor metastasis (Figure 2D and Supplementary Figure S1(B1,B2)), and immune activation (Figure 2E and Supplementary Figure S1(C1)). Additionally, the protein-protein interaction (PPI) analysis showed that PDE2A was a hub gene related to several key metabolic enzyme genes (Figure 2F): including ADSL (adenyl succinate lyase), AK3 (adenylate kinase 3), ADK (adenosine kinase), DCK (deoxycytidine kinase), APRT (adenine phosphoribosyl transferase), PDE6G (phosphodiesterase 6G), GUCY2D (guanylate cyclase 2D), ADCY4 (adenylate cyclase 4), GUCY1A2 (guanylate cyclase 1 soluble subunit alpha 2), and ADCY10 (adenylate cyclase 10). Hence, PDE2A may function in cell cycle, ECM organization, and ERK pathway in HCC.

### 3.3. PDE2A Expression Is Correlated with Immune Cell Infiltration in HCC

**Figure 3:**
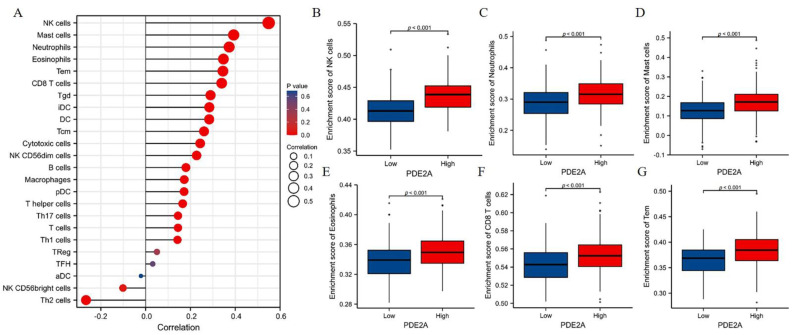
Correlation between PDE2A expression and Immune Cell Infiltration in HCC. (**A**) Relationships between infiltration levels of 24 immune cell types and PDE2A expression profiles by Spearman’s analysis. (**B**–**G**) The comparison of infiltration levels of most correlated immune cells between high- and low-PDE2A expression groups, including NK cells (**B**), neutrophils (**C**), mast cells (**D**), Eosinophils (**E**), CD8 T cells (**F**), and Tem (**G**). DCs, dendritic cells; aDCs, activated DCs; iDCs, immature DCs; pDCs, plasmacytoid DCs; Th, T helper cells; Th1, type 1 Th cells; Th2, type 2 Th cells; Th17, type 17 Th cells; Treg, regulatory T cells; Tgd, T gamma delta; Tcm, T central memory; Tem, T effector memory; Tfh, T follicular helper; NK, natural killer.

The GSEA results indicated that PDE2A was associated with immune activation. Thus, we further analyzed the correlation between PDE2A and the infiltration of immune cells in HCC. As shown in Figure 3A, PDE2A expression was positively correlated with nearly all immune cells investigated, including six that were strongly positively correlated with PDE2A expression (R ≥ 0.3, *p* < 0.001). Furthermore, we evaluated the infiltration levels of these six immune cells in the PDE2A groups: NK cells, Neutrophils, Mast cells, Eosinophils, CD8 T cells, and Tem cells (Figure 3B–G). These results were consistent with the observations from Figure 3A: HCC patients with low PDE2A expression harbored low immune cell infiltration activity.

### 3.4. Associations of PDE2A Expression and HCC Clinicopathological Variables

**Figure 4:**
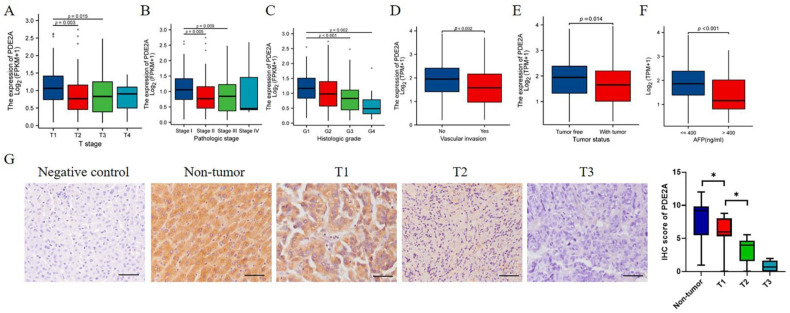
Associations of PDE2A expression and clinicopathological variables in HCC and immunohistochemical (IHC) staining of PDE2A in HCC samples. The associations between PDE2A expression with T stage (**A**), Pathologic stage (**B**), Histologic grade (**C**), Vascular invasion (**D**), Tumor status (**E**), and AFP level (**F**). (**G**) The representativeness images of PDE2A IHC staining results and the IHC score of PDE2A. Scale bar = 50 μm. AFP: alpha fetoprotein. * *p* < 0.05.

Next, we investigated the associations between PDE2A expression and clinicopathological characteristics of HCC patients. The expression level of PDE2A was negatively correlated with the T stage, pathologic stage, and histologic grade (Figure 4A–C). The protective role of PDE2A was also observed in high PDE2A expression HCC patients with no vascular invasion, tumor-free status, and low serum AFP levels (Figure 4D–F). The IHC results showed that PDE2A is highly expressed in non-tumor tissues and decreases with the T stage (Figure 4G).

### 3.5. Predictive Value of PDE2A Expression for HCC Patients’ Prognosis

**Figure 5:**
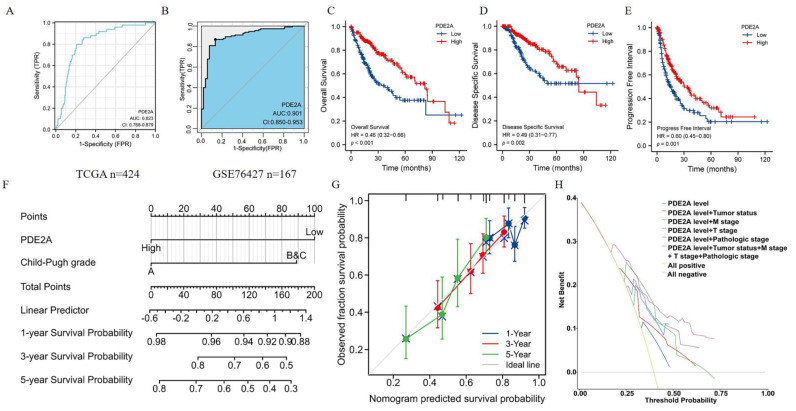
Predictive value of PDE2A expression for diagnosis and clinical outcomes in HCC patients. Receiver operating characteristic (ROC) curve analysis evaluating the performance of PDE2A for HCC diagnosis based on TCGA database (**A**) and GSE76427 database (**B**). (**C**–**E**) Kaplan–Meier analyses comparing overall survival (**C**), disease-specific survival (**D**), and progression-free interval (**E**) between high- and low-PDE2A expression groups. (**F**–**G**) Nomograms constructed to establish PDE2A expression-based risk scoring models for 1-, 3-, and 5-year overall survival. (**H**) Decision Curve Analysis (DCA) for the PDE2A expression, tumor status, M stage, T stage, and pathologic stage risk prediction models.

Further, we conducted ROC curve analyses to evaluate the prognosis predictive value of PDE2A in HCC and the area under the curve (AUC) value was 0.823 (Figure 5A). Further, we detected the diagnosis ability of PDE2A in GSE76427 database and the results showed that the AUC of PDE2A was 0.901 (Figure 5B). We also performed ROC curve analysis of other reported markers (AFP, GPC3, OPN3, VEGFA, ANG, IGF1, HGF, and MET) for comparison with PDE2A. The results showed that, except GPC3 and HGF, the AUC value of other markers is lower than PDE2A (Figure S2). Compared to the high PDE2A expression group, patients with low PDE2A expression had worse overall survival (OS), disease-specific survival (DSS), and progression-free interval (PFI). Furthermore, the multivariate Cox regression showed that the expression of PDE2A can be used as an independent prognosis marker for DSS and OS (Figure 5B–D) (Table S1). Meanwhile, to provide a quantitative approach predicting the prognosis of HCC patients, we construct nomograms using PDE2A and independent clinical risk factors. The calibration curve showed that the nomogram performs admirably in predicting the probability of survival in HCC patients (Figure 5E–F and Supplementary Figure S3A–D). Moreover, the 5-year Decision Curve Analysis (DCA) showed that PDE2A, combined with tumor status, pathological stage, M stage, and T stage could predict well the prognosis and survival of HCC patients (Figure 5G). Overall, PDE2A expression can be used as a superior factor for the long-term survival prediction of HCC patients.

### 3.6. Overexpression of PDE2A Inhibits the Proliferation, Colony Formation, Migration, and Invasion of HCC Cell Lines by Regulating the Mitochondrial Morphology and ATP Content

**Figure 6:**
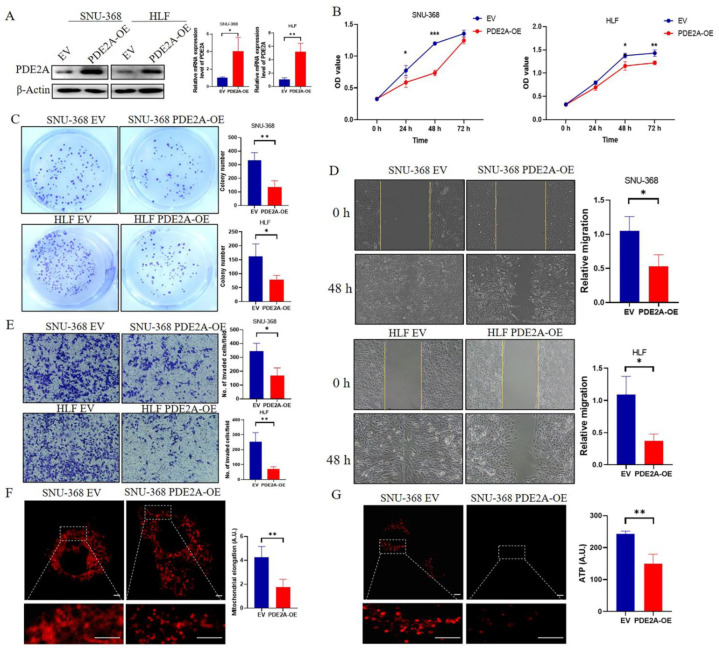
Overexpression of PDE2A inhibits the proliferation, colony formation, migration, and invasion of HCC cell lines by regulating the mitochondrial morphology and ATP content. (**A**) Western blot and RT-qPCR analysis for the protein and mRNA level in HCC cell lines SNU-368 and HLF under PDE2A-overexpressed treatment and empty vector (EV) as control. (**B**) Cell proliferation assay evaluated by MTS in HCC cell lines SNU-368 and HLF under PDE2A-overexpressed treatment and EV as control. (**C**) Colony formation assay in HCC cell lines SNU-368 and HLF under PDE2A-overexpressed treatment and EV as control. (**D**) Cell migration ability evaluated by scratch wound healing in HCC cell lines SNU-368 and HLF under PDE2A-overexpressed treatment and EV as control. (**E**) Cell invasion ability evaluated by Transwell Matrigel invasion assay in HCC cell lines SNU-368 and HLF under PDE2A-overexpressed treatment and EV as control. (**F**) Mitotracker staining of mitochondrial morphology in SNU-368 under PDE2A-overexpressed treatment and EV as control. (**G**) ATP probe detection of ATP content in SNU-368 under PDE2A-overexpressed treatment and EV as control. Scale bar = 0.25 μm. * *p* < 0.05; ** *p* < 0.01; *** *p* < 0.001.

Overexpressed PDE2A was verified in SNU-368 and HLF cells by RT-qPCR and Western Blot (Figure 6A). To verify the effects of PDE2A on HCC cell lines, we compared the cell proliferation rate between the EV and PDE2A-overexpressed groups by MTS assay. The results showed that PDE2A significantly inhibited the proliferation of HCC SNU-368 and HLF cell lines (Figure 6B). The colony formation assays also demonstrated that PDE2A overexpression inhibited the colony formation of HCC SNU-368 and HLF cell lines (Figure 6C). Cell migration and invasion experiments showed that PDE2A could inhibit the migration and invasion of HCC SNU-368 and HLF cell lines (Figure 6D,E). To explore the mechanism of PDE2A on HCC cells proliferation and migration, we performed Mitotracker staining to observe the mitochondrial morphology and the results showed that overexpression of PDE2A resulted in more fragmented mitochondria (Figure 6F), accompanied by ATP content decline (Figure 6G).

### 3.7. Knockdown of PDE2A Promotes the Proliferation, Colony Formation, Migration, and Invasion of HCC Cell Lines

**Figure 7:**
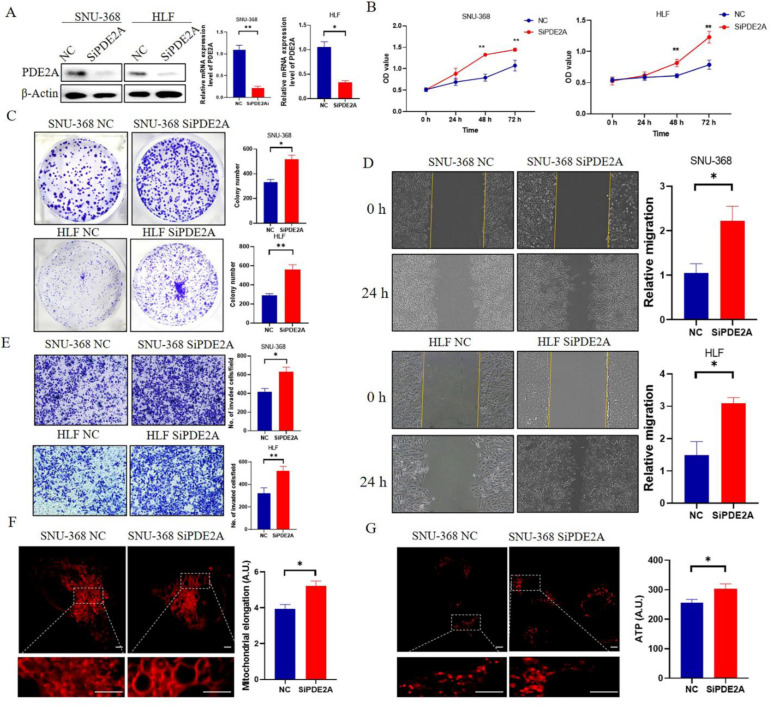
Knockdown of PDE2A promotes the proliferation, colony formation, migration, and invasion of HCC cell lines (**A**) Western blot and RT-qPCR analysis for the protein and mRNA level in HCC cell lines SNU-368 and HLF under PDE2A-siRNA treatment and negative control (NC). (**B**) Cell proliferation assay evaluated by MTS in HCC cell lines SNU-368 and HLF under PDE2A-siRNA treatment and NC as control. (**C**) Colony formation assay in HCC cell lines SNU-368 and HLF under PDE2A-siRNA treatment and NC as control. (**D**) Cell migration ability evaluated by scratch wound healing in HCC cell lines SNU-368 and HLF under PDE2A-siRNA treatment and NC as control. (**E**) Cell invasion ability evaluated by Transwell Matrigel invasion assay in HCC cell lines SNU-368 and HLF under PDE2A-siRNA treatment and NC as control. (**F**) Mitotracker staining of mitochondrial morphology in SNU-368 under PDE2A-siRNA treatment and NC as control. (**G**) ATP probe detection of ATP content in SNU-368 under PDE2A-siRNA treatment and NC as control. Scale bar = 0.25 μm. * *p* < 0.05; ** *p* < 0.01.

The Knockdown effects of PDE2A siRNA were verified by RT-qPCR and Western Blot and the knockdown rate is 70–80% (Figure 7A). MTS results showed that knockdown of PDE2A promotes HCC cells proliferation (Figure 7B) and the colony formation results showed that knockdown of PDE2A significantly increased the colony number (Figure 7C). Meanwhile, knockdown of PDE2A significantly promotes the migration and invasion of HCC cells (Figure 7D,E). The mitochondria tend toward more elongation in the PDE2A siRNA treated group than the negative control group (Figure 7F) and the ATP content increased in the PDE2A treated group also (Figure 7G).

## 4. Discussion

In most cases, the 5-year survival rate is below 40% when HCC patients are diagnosed at an advanced stage. Thus, the identification of novel molecular biomarkers would be crucial for HCC patients’ outcomes. In the present study, we first analyzed the expression levels of PDE2A in pan-cancer and showed that it was significantly downregulated in the vast majority of tumor tissues compared to non-tumor tissues, including HCC, except for two immune system tumors, DLBC and THYM. In HCC, a marked association of PDE2A expression with clinical characteristics, such as serum AFP levels, vascular invasion, histologic grade, and pathologic stage, was revealed. Based on this, we determined the predictive value of PDE2A for OS, DSS, and PFI in HCC patients. Compared to high PDE2A patients, low PDE2A patients exhibited worse OS, DSS, and PFI. Therefore, PDE2A expression is a protective factor in HCC. San-Zhong Li et al. found that targeted overexpression of PDE2A in glioma significantly repressed the stemness and decelerated glioma progression [13]. Then, Guihua Rao et al. found that PDE2A could be used as one of the stem cell index-associated genes to establish a cancer stem cell index-based model to stratify HCC risk and predict survival [4]. Lajos Pusztai et al. found that targeting of PDE2A and PDE5, the chemotherapy drug Exisulind in combination with capecitabine has anticancer activity in metastatic breast cancer patients [16]. Hao Ding et al. found that PDE2A might be a biomarker for early diagnosis and prognosis evaluation of cervical squamous cell carcinoma and endocervical adenocarcinoma patients [23]. miR-139 is located within the intron of PDE2A and its expression was significantly correlated with the expression of PDE2A. Kousuke Watanabe et al. found that overexpression of miR-139 suppressed invasion of lung cancer cells [24]. Moreover, Wong, C.C. et al. found that miR-139 is reduced in metastatic HCC patients and correlates with prognosis. Overexpression of miR-139 in HCC cells significantly reduced cell migration and invasion in vitro and the incidence and severity of lung metastasis from orthotopic liver tumors in mice [25]. There are also multiple reports found that PDE2A combined with other genes are a promising prognostic biomarker, such as for bladder cancer [26], metastatic colorectal cancer [27], and liver cancer [28]. However, the mechanism of PDE2A on tumor progression and prognosis remains unclear.

cAMP and cGMP are ubiquitous signaling molecules that function as second messengers in all domains of life. For example, cAMP activates protein kinase signaling, allowing cells to respond to external stimuli [29]. Unbalanced cAMP/cGMP upon PDE isoforms’ abnormal expression has been described in various cancer pathologies [30]. Specifically, PDE2A significantly regulates the growth and invasion of human breast cancer [31], malignant melanoma cells [12], osteosarcoma cells [32], and colon cancer [33]. Recently, researchers found that PDE2A also plays a key role in mitochondrial functions [9,10,34]. Herein, we found that PDE2A was a hub gene closely associated with ten metabolic enzymes and related to immunological activity in HCC. Besides, based on functional annotation of PDE2A-associated DEGs, PDE2A was closely associated with tumor metastasis pathways, including ECM organization, tumor progression pathways, such as ERK1 and ERK2 cascade, and digestive system development. The newly found functions of PDE2A made it a compelling target in HCC, in addition to the predicted prognostic potential. Actually, due to its extensive functions, PDE2A has already become a potential target for coronary atherosclerosis [35], psychiatric disease [36], and metabolic disease [37]. Moreover, our current in vitro experiments confirmed that PDE2A overexpression inhibited the proliferation, colony formation, migration, and invasion of HCC cells, demonstrating that PDE2A might be an effective target for HCC. While some researchers found that inhibition of another PDE isoform, PDE4B, suppressed colorectal cancer cell proliferation and survival. The contradictory results for different cells may be attributed to the main type PDE isoforms in different tissues being different, and there are unrecognized mechanisms of PDE isoforms on cell behaviors. For example, PDE2A is closely related with mitochondrial function, such as regulation of the activity of the respiratory chain [34], regulation of mitochondrial morphology [9], and inhibition of the phosphorylation of mitochondrial transcription factor A [11]. To explore the mechanism of PDE2A’s effect on HCC cells proliferation and migration, we detected the mitochondria morphology and found that PDE2A has a significant effect on HCC cells’ mitochondrial morphology and, further, on ATP content. Overexpression of PDE2A in HCC cell lines resulted in more fragmented mitochondria and thereby lower ATP content, and inhibition of PDE2A resulted in more elongated mitochondria and higher ATP content, which is consistent with a previous report in rat cardiac myocytes. ATP is a requisite in cells proliferation and migration [9]. Therefore, we thought PDE2A’s effect on HCC cells worked through regulating mitochondrial morphology and ATP content. Overall, the unique role of PDE2A makes it play a key role in influencing HCC prognosis.

In addition, we detected significantly positive correlations of PDE2A expression with Tem cells, CD8 T cells, Eosinophils, Mast cells, Neutrophils, and NK cells. Low PDE2A expression in HCC also showed weaker immune cell activities, resulting in a poor prognosis. CD 8 T cells are critical in initiating potent anti-tumor immunity [38]. The association between neutrophils and better prognosis for different cancers has been previously described [39]. Moreover, NK cells can kill cancerous cells, and NK cell-based cancer therapy has grown exponentially and currently constitutes a major area of immunotherapy innovation [40]. Therefore, low PDE2A expression in HCC seemed to impair anti-tumor immune responses, promote the escape of tumors from elimination, and finally accelerate tumorigenesis. Thomas K Hamilton et al. found that PDE influences immune stimulatory molecule-MHC class I-related chain A (MICA) expression, and thus influences NK cell function [41]. Additionally, cGMP is responsible for eliciting the innate immune response of the host only if it has not been degraded by some PDEs. Therefore, low expression of PDE2A in HCC may regulate the immune response through cGMP [42]. Moreover, cGMP-cAMP controlled cGAS-STING signaling pathway is a component of the innate immune system that functions to detect the presence of cytosolic DNA and, in response, trigger the expression of inflammatory genes that can lead to senescence or to the activation of defense mechanisms. Therefore, PDE2A expression is closely related with immune cell activities, and thus could influence HCC prognosis.

Notably, PDE2A expression in HCC has clinical significance. The ROC curve for PDE2A discrimination in HCC diagnosis had an AUC of 0.823, strongly suggesting that PDE2A was a convincing biomarker for HCC diagnosis. Although we obtained evidence demonstrating that PDE2A could be used as a prognosis marker and effective target for HCC, our study still has some limitations. First, our analyzed results were derived from RNA-seq data, and relative protein expression or downstream pathways need further investigation to verify their consistency with mRNA levels. Second, more in vitro and in vivo experiments must be performed to verify the signaling pathways obtained by GO and KEGG analysis. Finally, since most of the results were from TCGA, more datasets need to be analyzed to verify our results.

## 5. Conclusions

Herein, we demonstrated that low PDE2A expression might predict poor prognosis of HCC patients. Additionally, PDE2A might be a novel target for HCC.

## Data Availability

The data used to support the findings of this study are downloaded from https://portal.gdc.cancer.gov accessed on 27 March 2022, and https://www.ncbi.nlm.nih.gov/geo/query/acc.cgi?acc=GSE76427 accessed on 19 September 2022 or included within the article.

## Supplementary Materials

The following supporting information can be downloaded at: https://www.mdpi.com/article/10.3390/cells12010068/s1, Table S1: Cox regression analysis for clinical outcomes in HCC patients. Figure S1. Related to Figure 2. Gene Set Enrichment Analysis (GSEA) of differentially expressed mRNAs between high- and low-PDE2A expression groups except for the representative figures showed in Figure 2. Figure S2. Receiver operating characteristic (ROC) curve analysis of reported markers (A) AFP; (B) GPC3; (C) OPN3; (D) VEGFA; (E) ANG; (F) IGF1; (G) HGF; and (H) MET for HCC diagnosis based on TCGA database. Figure S3. Related to Figure 5. Nomograms constructed to establish PDE2A expression-based risk scoring models for 1-, 3-, and 5-year disease-specific survival (A) and progression-free interval (C). Calibration curves for 1-, 3-, and 5-year patient disease-specific survival (B) and progression-free interval (D).
