# Supplementary material for: Low Expression of Phosphodiesterase 2 (PDE2A) Promotes the Progression by Regulating Mitochondrial Morphology and ATP Content and Predicts Poor Prognosis in Hepatocellular Carcinoma
Source: Cells. 2022 Dec 23;12(1):68. doi: 10.3390/cells12010068 (PMC9818237; doi:10.3390/cells12010068)
Supplement: Supplementary file 1 [file cells-12-00068-s001.zip › cells-1980401-supplementary.pdf]

Supplementary materials:

Table S1. Cox regression analysis for clinical outcomes in HCC patients.

Characteristics	HR for overall survival (95% CI)		HR for progression-free interval (95% CI)		HR for disease-specific survival (95% CI)	
	**Univariate**	**Multivariate**	**Univariate**	**Multivariate**	**Univariate**	**Multivariate**
PDE2A ((high vs. low))	**0.521***	**0.505**	**0.655**	0.925	**0.503**	**0.243**
Age (>60 vs. ≤60 years)	1.205		0.960		0.846	
Tumor status (With tumor vs Tumor free)	**2.317***	**1.902**	**11.342***	**14.199***	775790759.389	
Histologic grade (G3&G4 vs G1&G2)	1.091		1.152		1.086	
Pathologic stage (Stage III&Stage IV vs Stage I&Stage II)	**2.504***	1.573	**2.201***	2.214	**3.803***	0.866
T stage (T3&T4 vs T2&T1)	**2.598***	1.590	**2.177***	0.671	**3.639***	3.294
N stage (N1 vs N0)	2.029		1.370		3.612	
M stage (M1 vs M0)	**4.077**	1.373	**3.476**	1.604	**5.166**	3.318
BMI (>25 vs <=25)	0.798		0.936		0.826	
AFP(ng/ml) (>400 vs <=400)	1.075		1.045		0.867	
Vascular invasion (Yes vs No)	1.344		**1.676**	1.488	1.277	
Fibrosis ishak score (3/4&5/6 vs 0&1/2)	0.740		1.209		0.660	
Adjacent hepatic tissue inflammation (Mild&Severe vs None)	1.194		1.238		1.403	
Child-Pugh grade (B&C vs A)	1.643		1.395		**2.560**	**4.156**
Prothrombin time (>4 vs <=4)	1.335		1.100		**1.778**	1.476
Residual tumor (R1&R2 vs R0)	1.604		1.513		1.678	
Albumin(g/dl) (>=35 vs <35)	0.897		0.911		1.148	
Gender (Male vs Female)	0.793		0.982		0.813	

*P< 0.05; **P< 0.01; ***P< 0.001.

Figure S1.

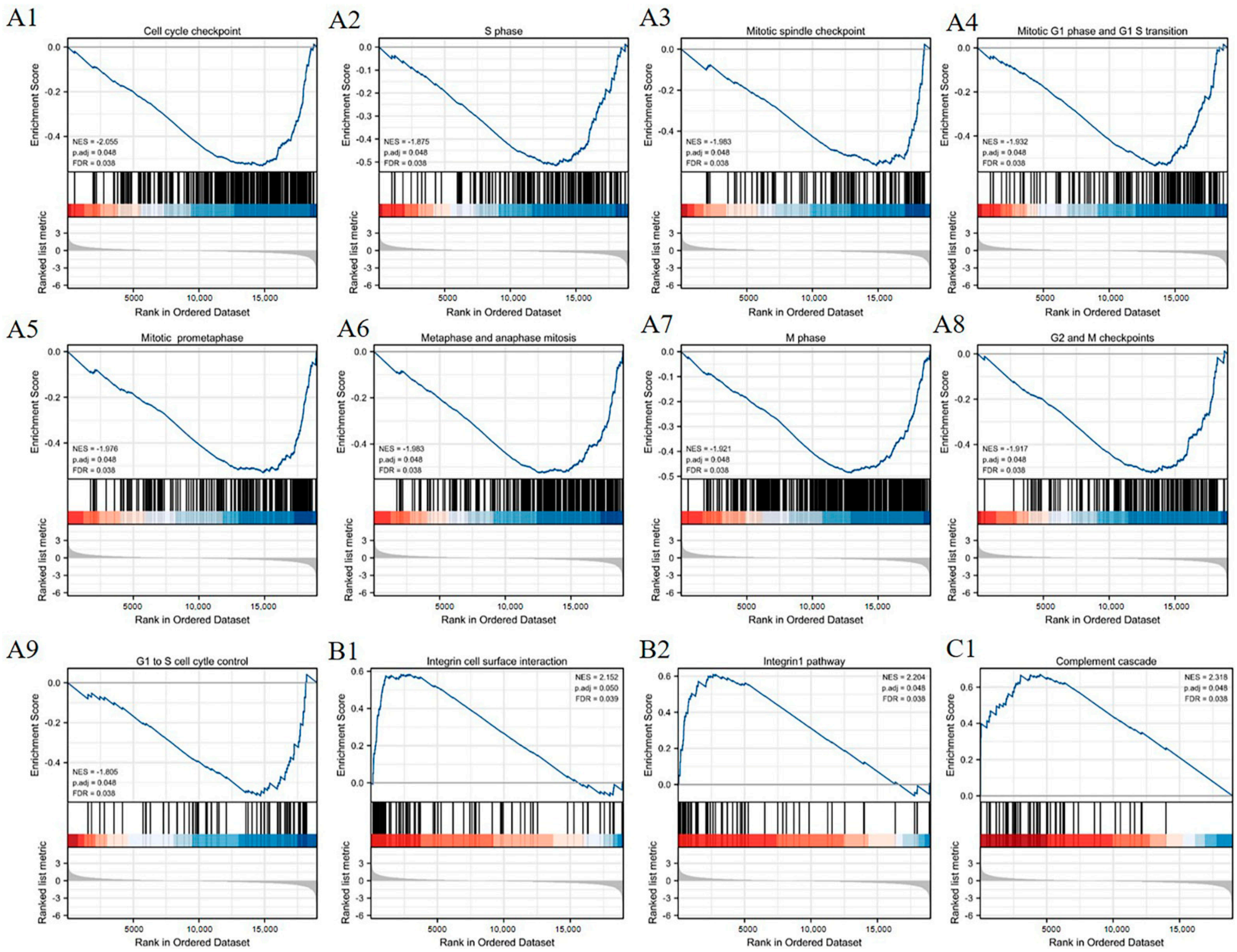

Figure S1. Related to Figure 2. Gene Set Enrichment Analysis (GSEA) of differentially expressed mRNAs between high- and low-PDE2A expression groups except for the representative figures showed in Figure 2.

Figure S2.

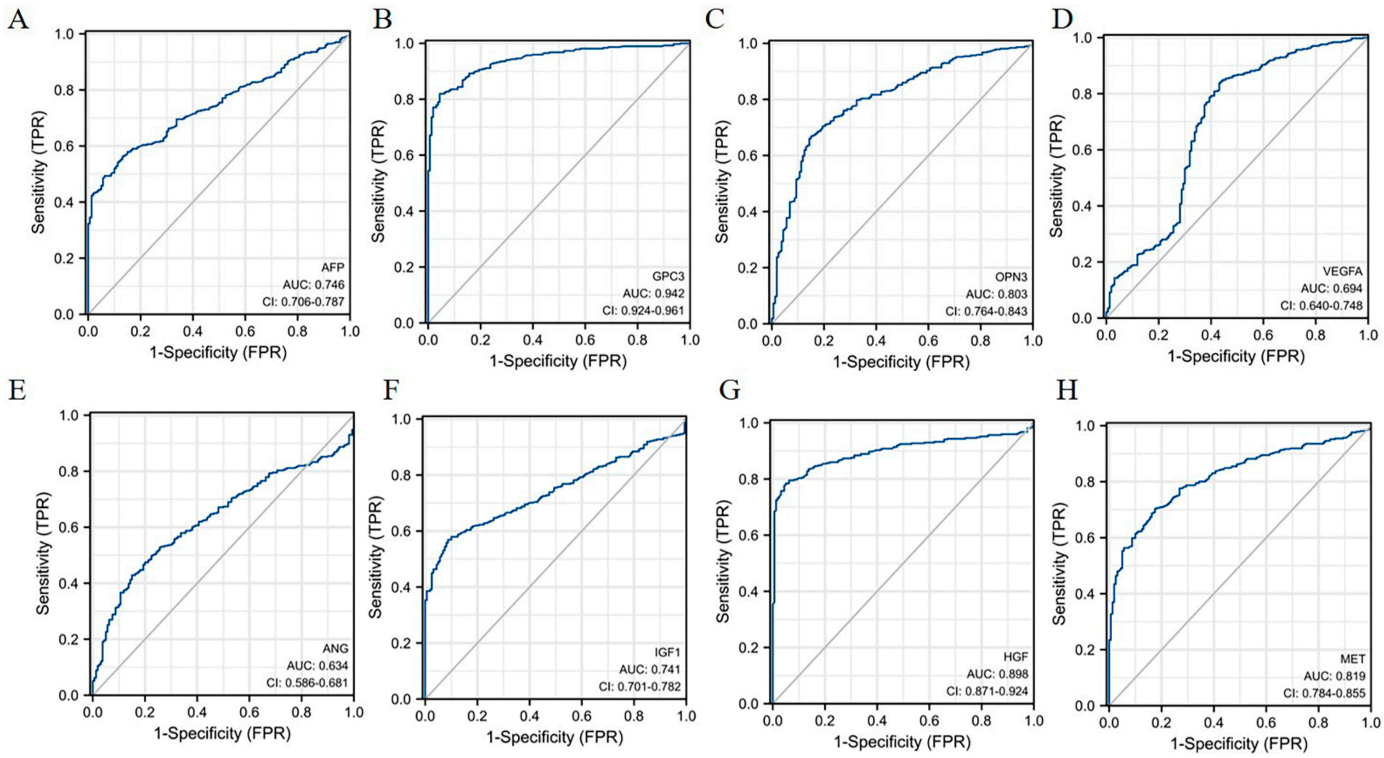

Figure S2. Receiver operating characteristic (ROC) curve analysis of reported markers (A)AFP; (B)GPC3; (C)OPN3; (D)VEGFA; (E)ANG; (F)IGF1; (G)HGF; and (H)MET for HCC diagnosis based on TCGA database.

Figure S3.

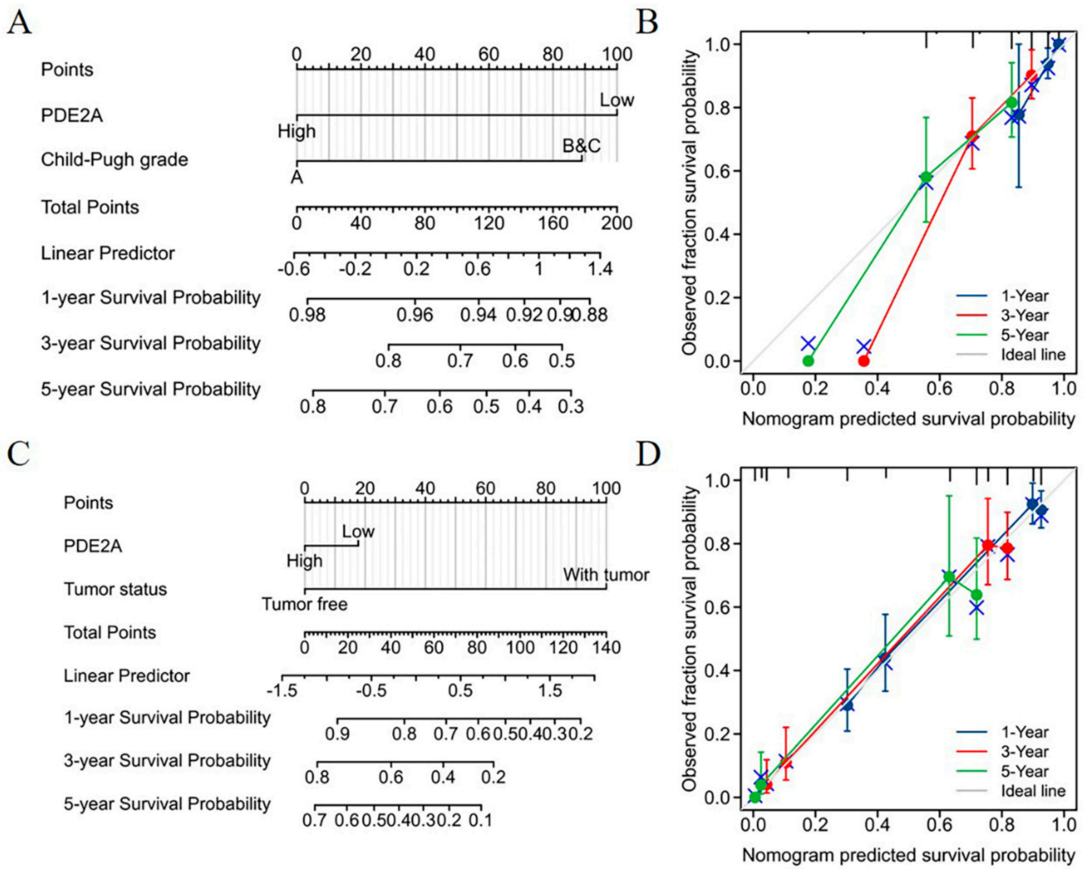

Figure S3. Related to Figure 5. Nomograms constructed to establish PDE2A expression-based risk scoring models for 1-, 3-, and 5-year disease-specific survival (A) and progression-free interval (C). Calibration curves for 1-, 3-, and 5-year patient disease-specific survival (B) and progression-free interval (D).
